# A 15-cm Adrenal Incidentaloma Suggested on Upper Endoscopy and Resected Laparoscopically

**DOI:** 10.7759/cureus.29979

**Published:** 2022-10-06

**Authors:** Sami Saleh, Nagham Bazzi, Hadeel Shamma, Nazem Nouraldin Haj, Mhd Firas Safadi

**Affiliations:** 1 General Surgery, Syrian Specialist Hospital, Aleppo, SYR; 2 Medicine, Lebanese University, Beirut, LBN; 3 Pathology, Klinikum Chemnitz, Chemnitz, DEU; 4 Visceral, Thoracic and Vascular Surgery, University Hospital Carl Gustav Carus, Technische Universität Dresden, Dresden, DEU

**Keywords:** adrenal myelolipomatous metaplasia, myelolipomatous changes within adrenocortical adenoma, duodenal compression, upper endoscopy, laparoscopic adrenalectomy, adrenal tumors, adrenal disease, adrenal adenoma, incidentaloma, adrenal incidentalomas

## Abstract

Incidentalomas are asymptomatic adrenal tumors that are discovered on investigations performed for other reasons. Classically, these tumors are found on computed tomography of the abdomen. This article describes an incidentaloma that was discovered on upper gastrointestinal endoscopy performed before a bariatric operation and caused an extrinsic compression of the first and second parts of the duodenum. Further investigations showed a 15-cm non-functional adrenal tumor. The patient was treated successfully with laparoscopic adrenalectomy. The histological examination showed a benign adrenocortical adenoma with myelolipomatous changes. The article highlights the fact that incidentalomas may not only be discovered on imaging modalities but may also show up in other diagnostic methods such as endoscopy.

## Introduction

Adrenal incidentalomas are asymptomatic tumors that are usually detected on computed tomography (CT) of the abdomen performed for other reasons [1]. These tumors are found in up to 5% of abdominal CT examinations and reach a peak in the sixth decade of life [2].

Incidentalomas greater than 1 cm need a diagnostic workup to exclude hormonal activity [1]. Small non-functional tumors can be followed with no need for treatment. Surgical resection should be offered in lesions greater than 4 cm [1]. The minimally invasive approach is increasingly becoming an attractive therapeutic option even for large tumors [2].

In this case, we present an incidentaloma in a young female that was suggested on upper gastrointestinal endoscopy during preoperative preparation for a bariatric operation. To our knowledge, this is the first described incidentaloma that was detected on endoscopy.

## Case presentation

A 29-year-old woman presented to the surgical clinic seeking a medical opinion for bariatric surgery. Her past medical history was not significant and her surgical history was limited to two cesarean sections. The family history included arterial hypertension and diabetes mellitus type 2.

The physical examination was unremarkable. The patient’s BMI was 42.2 kg/m^2^ (height 1.63 meters, weight 112 kilograms). The blood pressure and the laboratory tests were within the normal limit. A further diagnostic study was initiated.

**Figure 1:**
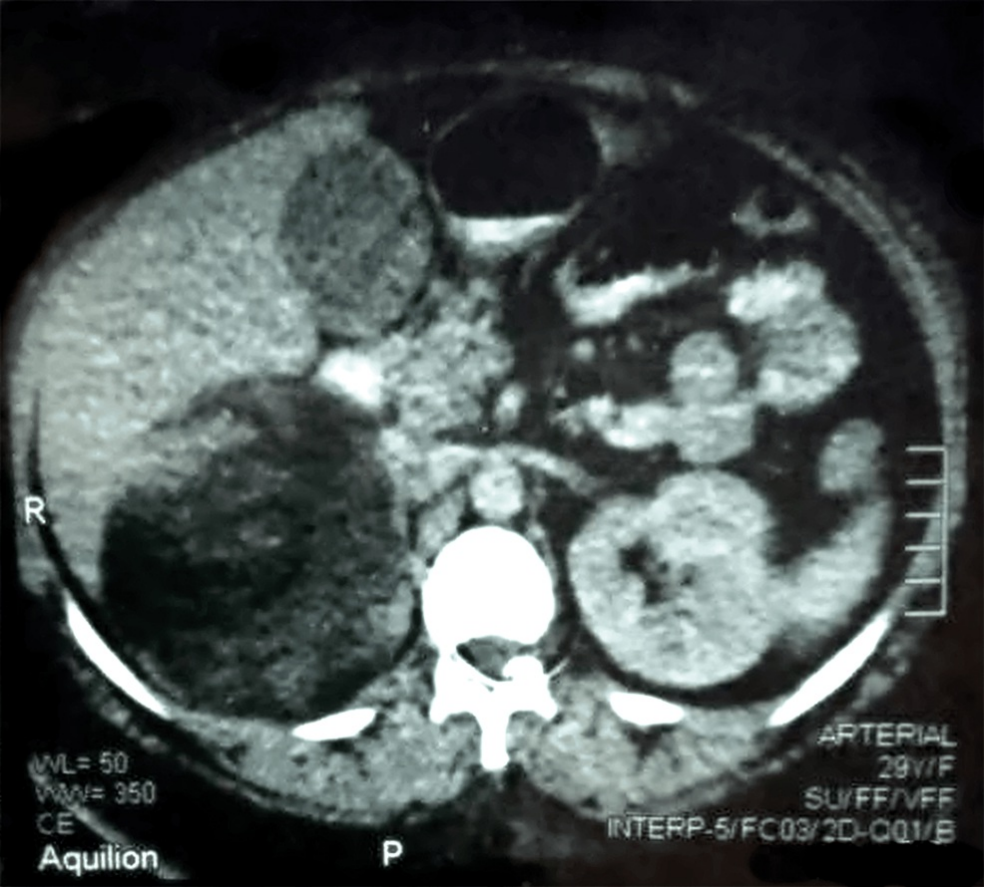
Computed tomography of the abdomen, transverse section with oral and intravenous contrast enhancement A large inhomogeneous lesion is seen dorsal to the liver. The mass measures up to 15 cm and displaces the liver anteriorly and the neighboring organs medially. The study shows no evidence of local invasion, enlarged lymph nodes, contralateral masses, or liver metastases.

In the esophagogastroduodenoscopy, the gastric and duodenal mucosa showed no abnormalities, but the first and second parts of the duodenum were collapsed, apparently due to external compression from the right side. Abdominal ultrasound revealed a large subhepatic tumor with downward displacement of the right kidney. For more evaluation, computed tomography (CT) and magnetic resonance imaging (MRI) of the abdomen were performed. These showed a right adrenal mass up to 15 cm in diameter with no evidence of local infiltration, vascular invasion, or metastatic lesions (Figure 1).

With an adrenal incidentaloma as a working diagnosis, we initiated a comprehensive laboratory study in cooperation with our endocrinologists to detect possible hormonal activity. The values of glucocorticoids, catecholamines, and androgens were all at the expected level, which could rule out Cushing’s syndrome, pheochromocytoma, and androgen-secreting tumors, respectively. Accordingly, the diagnosis of nonfunctional adrenal incidentaloma was established. Since the preoperative imaging showed no signs of malignancy, we made the indication for laparoscopic adrenalectomy and obtained the patient’s consent for a possible conversion to open surgery.

**Figure 2:**
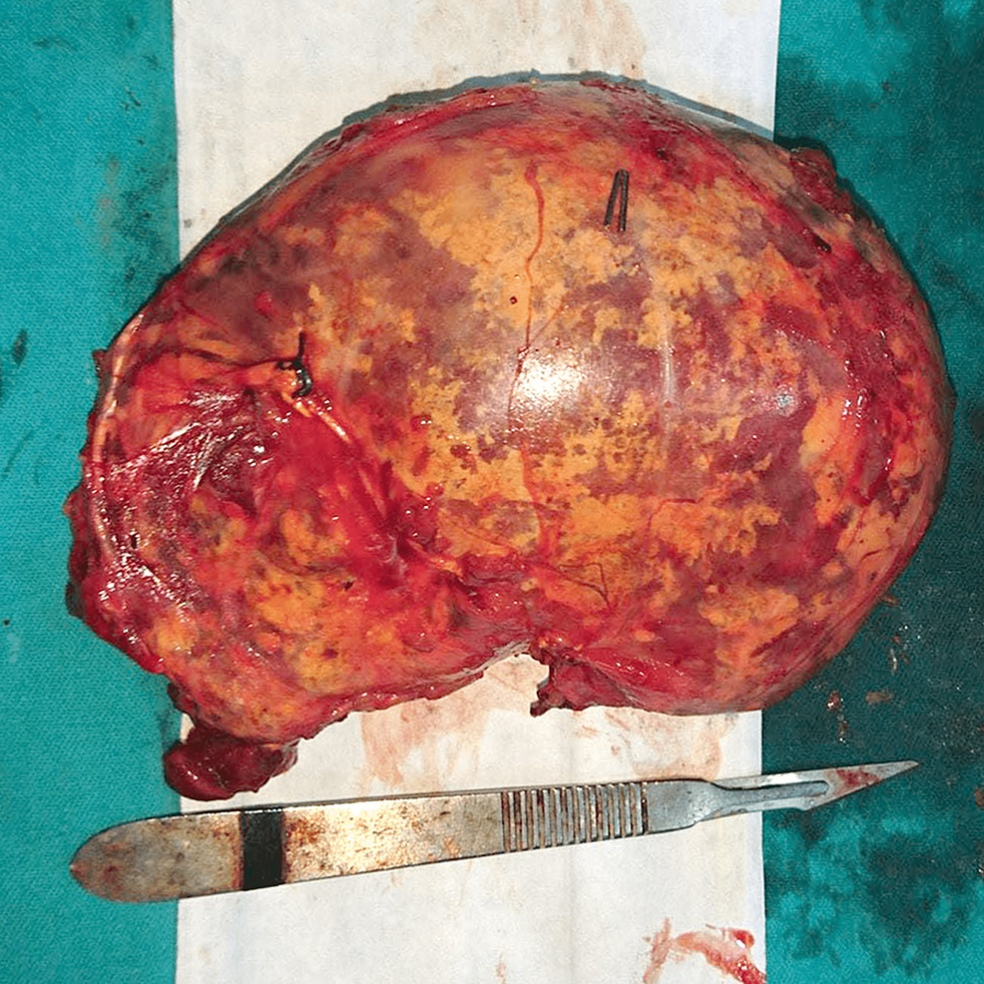
Gross examination of the resected specimen showing the large encapsulated mass The tumor measured 15 x 11 x 8 cm and weighed 410 grams. Some ligated blood vessels can be seen coursing on the smooth surface. Note also the visible, yellowish lipomatous components of the tumor as seen through the thin capsule.

The procedure could be performed using transperitoneal laparoscopy in the left lateral position. Four trocars were used: three trocars below the costal margin and a fourth trocar on the lateral margin of the rectus muscle at the umbilical level. The large mass was retrieved after expansion of the last-mentioned port site. The duration of surgery was about 210 minutes and the intraoperative blood loss was less than 200 ml. The patient could be discharged home after four days. The final histological examination showed an adrenocortical adenoma with islands of myelolipoma; no malignancy was detected (Figure 2).

## Discussion

In this case report, we illustrate an uncommon presentation of a large adrenal tumor that was incidentally suggested on upper gastrointestinal endoscopy in a young woman. Adrenal tumors are relatively common neoplasms that may be found in up to 7-10% of the population on autopsy [2,3]. The vast majority of these tumors are non-functional, with a prevalence of 85% [4]. Adrenal tumors may be asymptomatic, may trigger symptoms related to local compression, or may cause systematic symptoms related to hormonal hypersecretion [4].

The term “incidentaloma” dates back to 1982 when Geelhoed and Druy initially used it to describe asymptomatic adrenal masses discovered during investigations for other reasons, usually abdominal computed tomography (CT) [5]. The frequent use of abdominal CT during the last decades has increased the incidence of adrenal tumors by 10-fold [6], and the prevalence of these masses on abdominal CT ranges from 0.3-5.1% [2]. The incidence of incidentalomas increases with age with a peak in the sixth decade. They are rarely seen before the age of 30 as in the patient described in this case [2]. A cut-off of 1 cm is used for the decision of additional diagnostic workup to exclude hormonal hypersecretion syndromes [1].

Incidentalomas are classically discovered on the CT scan of the abdomen. In principle, any asymptomatic adrenal tumor that is found in investigations performed for other reasons can be designated as an incidentaloma, including the tumor found in our case. External compression can be detected on upper or lower endoscopy and may be confused with intramural lesions [7]. Kumar et al. described 79 patients with extrinsic compression detected on upper endoscopy. Most lesions originated from the liver or pancreas, but none of the patients had an adrenal lesion [7]. To the best of our knowledge, no previous reports described an adrenal incidentaloma detected on endoscopy.

The vast majority of incidentalomas are adrenocortical adenomas [2]. Myelolipoma is one of the other variants, which is seen in only 3% of cases [8]. The combination of the two types is considered a rarity with about 20 cases described in the literature so far [8]. Such benign tumors are described as adrenocortical adenomas containing foci of adipose tissue intermixed with bone marrow elements, as was reported in our case. Firat et al. described this appearance as "myelolipomatous changes within adrenocortical adenoma" [9] and Anbardar et al. described it as "myelolipomatous metaplasia" [8].

Most adrenal incidentalomas are between 3 and 3.5 cm in largest diameter [10]. The largest adrenal incidentaloma reported in the literature measured 23 cm [11] while the largest documented symptomatic adrenal tumor was as large as 32 cm [10]. The indications for laparoscopic resection of adrenal masses are still controversial. Many authors argued that an open approach should be adopted in tumors greater than 10-12 cm [12]. Zografos et al. described a laparoscopic removal of 14 cm adrenal tumor [13]. The tumor in our case was 15 cm in diameter, which is, to our knowledge, the largest adrenal tumor resected laparoscopically. We recommend laparoscopic or robotic-assisted adrenalectomy, either transperitoneal or retroperitoneal, even in larger adrenal tumors, with specific contraindications including suspicion of malignancy, local infiltration, or insufficient expertise or resources [2].

The benefits of the minimally-invasive approach were proved in many previous works. In their study of 1683 adrenalectomy patients, Shahait et al. showed that laparoscopic adrenalectomy was associated with lower morbidity and mortality, shorter length of stay, and less blood loss compared with the open approach [14]. In their retrospective analysis, Mohammed et al. proved that there is no priority of the open approach in adrenal tumors that are 5 cm or larger concerning complication rate, length of stay, mortality, or oncologic outcomes. Accordingly, they recommended laparoscopic adrenalectomy for all adrenal tumors regardless of their size [15]. Robotic-assisted resection was also recommended in more difficult cases or larger tumors, as it may provide the needed dexterity for handling such tumors [16]. Therefore, it is no wonder that the use of minimally invasive adrenalectomy is on the rise, with trend analysis showing an increase of eight-fold over a period of 20 years [14].

## Conclusions

Adrenal incidentalomas are most commonly discovered on abdominal computed tomography, but they may be also detected by chance in other investigations such as endoscopy. All patients with incidentalomas greater than 1 cm should have a proper diagnostic workup. Small, non-functional, benign-appearing tumors do not need treatment. Surgery is recommended in tumors larger than 4 cm, with minimally invasive surgery being recommended based on the patient’s anatomy, the characteristics of the tumor, the skill of the surgeon, and the available resources. Most incidentalomas are benign, with adrenocortical adenoma being the most common variant. Associated myelolipomatous changes are one of the rare manifestations than can be found in incidental adenomas.

## References

[1] Fassnacht M, Arlt W, Bancos I (2016). Management of adrenal incidentalomas: European Society of Endocrinology Clinical Practice Guideline in collaboration with the European Network for the Study of Adrenal Tumors. Eur J Endocrinol.

[2] Sherlock M, Scarsbrook A, Abbas A, Fraser S, Limumpornpetch P, Dineen R, Stewart PM (2020). Adrenal incidentaloma. Endocr Rev.

[3] Lopez D, Luque-Fernandez MA, Steele A, Adler GK, Turchin A, Vaidya A (2016). "Nonfunctional" adrenal tumors and the risk for incident diabetes and cardiovascular outcomes: a cohort study. Ann Intern Med.

[4] Singh PK, Buch HN (2008). Adrenal incidentaloma: evaluation and management. J Clin Pathol.

[5] Geelhoed GW, Druy EM (1982). Management of the adrenal "incidentaloma". Surgery.

[6] Bancos I, Prete A (2021). Approach to the patient with adrenal incidentaloma. J Clin Endocrinol Metab.

[7] Kumar K, Patel H, Mehershahi S (2019). Clinical relevance of endoscopically identified extrinsic compression of the oesophagus and stomach. BMJ Open Gastroenterol.

[8] Anbardar MH, Soleimani N, Nikeghbalian S, Mohebbi M (2021). Adrenocortical adenoma with myelolipomatous metaplasia: a potential diagnostic pitfall: a case report and review of the literature. J Med Case Rep.

[9] FIRAT C, Eryiğit S, Yener S (2019). Myelolipomatous changes within adrenocortical adenoma. Cukurova Med J.

[10] Li B, Guo Q, Yang H, Guan J (2013). Giant non-functional adrenal adenoma: a case report. Oncol Lett.

[11] Kasperlik-Załuska AA, Otto M, Cichocki A (2008). Incidentally discovered adrenal tumors: a lesson from observation of 1,444 patients. Horm Metab Res.

[12] Zografos GN, Korkolis D, Georgoutsos P (2002). Giant myelolipoma of the right adrenal gland. Int J Clin Pract.

[13] Zografos GN, Farfaras A, Vasiliadis G (2010). Laparoscopic resection of large adrenal tumors. JSLS.

[14] Shahait A, Saleh K, Weaver D, Mostafa G (2022). Two decades’ outcomes and trends of adrenalectomy for benign pathologies in veterans. Surg Laparosc Endosc Percutan Tech.

[15] Mohammed A, Amine H, Atiq SE (2018). Applicability and outcome of laparoscopic adrenalectomy for large tumours. Pan Afr Med J.

[16] Nomine-Criqui C, Germain A, Ayav A, Bresler L, Brunaud L (2017). Robot-assisted adrenalectomy: indications and drawbacks. Updates Surg.

